# Molecular Dynamics of Cytokine Interactions and Signalling of Mesenchymal Stem Cells Undergoing Directed Neural-like Differentiation

**DOI:** 10.3390/life12030392

**Published:** 2022-03-08

**Authors:** Jerran Santos, Penelope V. Dalla, Bruce K. Milthorpe

**Affiliations:** Advanced Tissue Engineering and Stem Cell Biology Group, School of Life Sciences, University of Technology Sydney, Sydney 2007, Australia; penelope.dalla@uts.edu.au (P.V.D.); bruce.milthorpe@uts.edu.au (B.K.M.)

**Keywords:** neural, differentiation, mesenchymal stem cells, adipose-derived stem cells, secretions, extracellular vesicles, cytokines, multiplex assay, interaction networks

## Abstract

Mesenchymal stem cells are a continually expanding area in research and clinical applications. Their usefulness and capacity to differentiate into various cells, particularly neural types, has driven the research area for several years. Neural differentiation has considerable usefulness. There are several successful differentiation techniques of mesenchymal stem cells that employ the use of small molecules, growth factors and commercially available kits and supplements. Phenotyping, molecular biology, genomics and proteomics investigation revealed a wealth of data about these cells during neurogenic differentiation. However, there remain large gaps in the knowledge base, particularly related to cytokines and how their role, drive mechanisms and the downstream signalling processes change with their varied expression throughout the differentiation process. In this study, adult mesenchymal stem cells were induced with neurogenic differentiation media, the cellular changes monitored by live-cell microscopy and the changes in cytokine expression in the intracellular region, secretion into the media and in the extracellular vesicle cargo were examined and analysed bioinformatically. Through this analysis, the up-regulation of key cytokines was revealed, and several neuroprotective and neurotrophic roles were displayed. Statistically significant molecules IFN-G, IL1B, IL6, TNF-A, have roles in astrocyte development. Furthermore, the cytokine bioinformatics suggests the Janus Kinase/Signal Transducer and Activator of Transcription (JAK/STAT) pathway is upregulated, supporting differentiation toward an astroglial lineage.

## 1. Introduction

Mesenchymal stem cells (MSCs) have been an in-vogue research topic for more than a decade [1,2]. Due to their ease of access through various adult tissues, they bypass the ethical dilemma of embryonic stem cells and modified status of induced pluripotent stem cells [3]. While those cells certainly have their advantages, MSCs have the benefit of being non-genetically modified as well as having a clinical readiness to maintain their autologous or allogenic status which is dependent on use [4]. In research, MSCs, are proven to be useful for investigating maturation and differentiation into varied progeny cell types [5,6]. The most widely investigated is neural lineage differentiation [7]. This is primarily due to the enormous clinical applicability for a range of disorders from neurodegenerative, trauma, ischemia, and congenital and regenerative applications [4].

Neural differentiation can be initiated in MSCs by a range of published methods providing a range of progeny cells dependent on the concentration and timeframes used [8,9]. Some applications use individual induction chemicals, such as valproic acid [10,11], retinoic acid [12], beta-mercaptoethanol [8] and dimethylsulfoxide [13], in a basal media and have had a varied range of success in priming or inducing early stages of differentiation in short periods of time. The application of growth factors such as brain-derived neurotrophic factor, fibroblast growth factor or epithelial growth factor are used in longer timeframes and have also achieved varying success [14,15]. The use of complex cocktails or commercially available kits provides a mixture of the above, including supplemental ingredients to support the multistep process [16,17,18]. Whilst there is still no definitive process or perfect media type, some offer greater advantages or outcomes. The use of commercial kits targets a unified and user-friendly system that is increasing in popularity. However, the molecular processes activated and progressed are still not completely understood across any media type. Ranges of differentiation are achieved with produced cells attaining morphological appearances, surface markers as well as a host of proteomic and transcriptomic data to support the neurogenic progression. However, while the research slowly unravels the complexities of the neural differentiation of MSCs, several unknowns still exist. One of these unknowns is the complex role cytokines play in MSC neural differentiation [19].

Cytokines are pleiotropic signalling molecules produced by almost all cells in the body that assist cell to cell communication [20]. They are a diverse group made up of interleukins, chemokines, growth factors and tumour necrosis factors [20]. They are known to be highly multifunctional from communication, response to external stimuli, controlling inflammatory states as well as controlling the migration of cells to sites of infection or injury [21]. Their multifunctionality can be manipulated by their expression levels, sites acted upon, available surface receptors, types of cells producing them, types of cells affected, up and downstream signalling and importantly, their location. At the cellular level, cytokines may have a particular function intracellularly and other functions extracellularly [21,22]. Intracellular cytokines may enact their range of effects within the cell they were expressed from, they may act upon surface receptors instigating a variety of outputs or they may be delivered between cells as extracellular vesicle cargo [23,24]. This transport is important as cytokines cannot passively cross the lipid bilayer. Extracellular vesicles (EVs) are formed from the outward budding and fission of the plasma membrane [25]. This process is now known to be a controlled cellular mechanism for compiling and packaging specific cargo such as proteins, nucleic acids, metabolites and cytokines [26]. Their roles in stem cell biology are also on an upward trend in research to elucidate their biological roles in cell-to-cell communication. Furthermore, there is a growing interest and the translational application of EV particles to treat various afflictions [25].

Cytokines play critical roles in the developing brain and central nervous system, promoting cellular migration, maturation and differentiation into specific cell types such as neurons and glial cells [27,28,29]. Their postulated role as key modulators of neurogenesis is explored in their control of signalling cascades and receptor activation that leads to downstream gene regulation, which is vital in differentiation and neural support [19]. As such, their location is an essential element in cellular communication, with the three main areas being intracellular, secreted or extracellular vesicle cargo. Analysing the cytokine content in these three locations in stem cells and their differentiated progeny may provide greater insight into their expression and subsequent roles in MSC differentiation.

As such, the aim of this study was to comprehensively characterise MSC cytokines as they undergo neural differentiation. The primary adipose-derived stem cells (ADSCs) were treated with two commercial media formulations, neurobasal media B27 and terminal differentiation media (TDM), and examined the cytokine expression changes between the undifferentiated and the treated cells. Furthermore, the secreted material and the extracellular vesicles were also isolated from each cell type for a comprehensive investigation. Using live cell microscopy, the morphological changes were tracked through temporal differentiation selected timepoints. The cytokines and chemokine analysis were completed using a sandwich ELISA multiplex analysis to quantify 27 separate cytokines in each sample type. The post hoc analysis investigated the roles the measured molecules have in signalling cascades and ontological analyses to gain insights into their role in neural differentiation.

## 2. Results

### 2.1. Live Cell Microscopy during Differentiation of ADSCs

In Figure 1:

(A) The characteristic semi-fibroblastic morphology of the control ADSCs with short tripolar regions and uniform cytoplasmic regions is presented. The overall population grows in an irregular pattern across the culture vessel;
(B) Control ADSCs grown for 28 days in the control media are presented. This shows a unique view of the ADSCs in the Day 0 control compared to the day 28 B27 and TDM cultures. The ADSCs at day 28 appear to be highly stressed with overgrowth, cell death and areas of senescence;
(C) ADSCs treated with the B27 media additive at 14 days post-treatment show a marked difference from the controls, with cells exhibiting the elongation of cells with bipolar region extensions reaching between cells. The population displays a somewhat irregular growth pattern with some directional polarity appearing in the righthand side of the image;
(D) The ADSCs 28 days post B27 treatment are presented with a vastly different population compared to the controls, where the majority of the cells have now adopted the elongated bipolar shape with a dense central body. The cells now extend between neighbouring cells. In addition, the population have adopted a much more directional growth pattern resembling fingerprint rivers and bifurcations;
(E) TDM treatment in continuous culture at day 14 shows relatively similar changes as the B27, with cell projections and elongation with fine pseudopodia-like structures with extension formation;
(F) Post-TDM treatment, the cells show loosely directional growth such as the B27 at day 14; the cellular morphology is also a bipolar shape; however, the cells appear to be wider and more diffused, with the central body being flatter and wider than the B27 at 28 days;
(G) The cell count from the control ADSCs and the final timepoints for B27 and TDM treated ADSCs are presented. Day 14 B27, TDM and day-28 control ADSCs were not performed as the former were in a continuous culture and the latter were not useful for any further analysis. The B27 and TDM treated ADSCs show a statistically significant increase in cell number compared to the controls.

**Figure 1:**
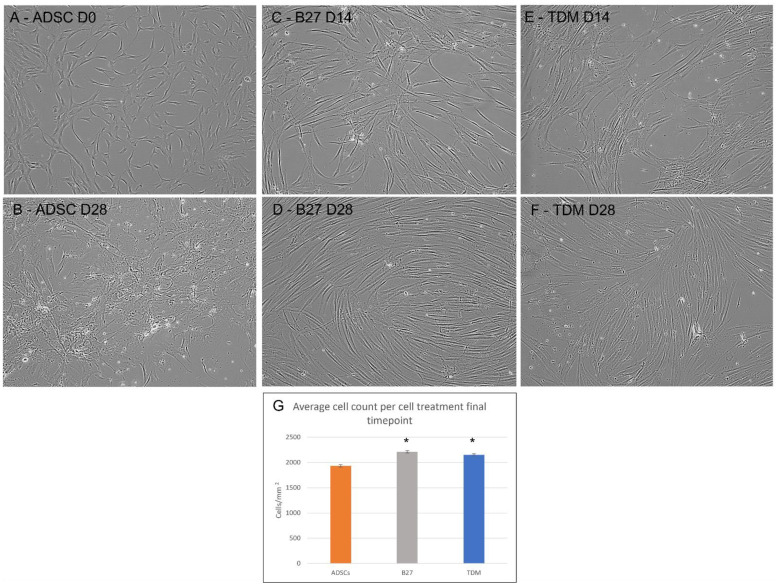
Microscopy of temporal differentiation of adult mesenchymal stem cells temporal differentiation with B27 and TDM media captured at 10× magnification. (**A**) ADSCs Control Day 0, (**B**) ADSCs Day 28, (**C**) B27 treated ADSCs Day 14, (**D**) B27 treated ADSCs Day 28, (**E**) TDM treated ADSCs Day 14, (**F**) TDM treated ADSCs Day 28, (**G**) Cell count for ADSC control (orange), B27 day 28 (grey) and TDM day 28 (blue). A Student’s *t*-test was performed between all cell treatments triplicates using a single tail homoscedastic test where the statistically significant *p*-value is presented as * < 0.05.

### 2.2. Cytokine Content from EVs, Cells and Secretion Heatmap and Dendrogram Clustering

**Figure 2:**
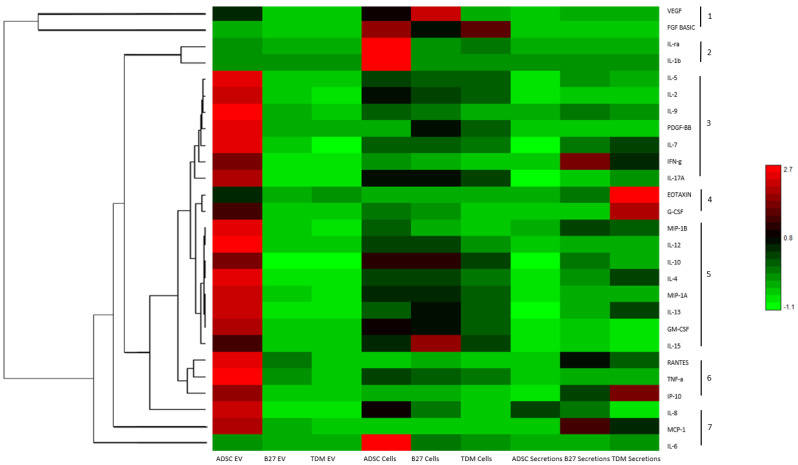
Heatmap and dendrogram of Bioplex quantified cytokines from cells, EVs and secretions derived from control ADSCs, B27 treated cells and TDM treated cells. Log10 scale where relatively red is high and green is low. The dendrogram represents hierarchical Euclidean clustering of cytokines across measured samples with 7 distinct groups.

ADSCs treated with B27 and TDM media produced distinct morphological traits by the final time point. At this point, the cellular material, EVs and secretions were collected for further molecular analysis by examining cytokine expression and content in each sample type. The EV population included those vesicles that pellet at 20,000× *g*. Cytokine levels from each sample were analysed using the Bioplex 27-plex human pro-inflammatory kit to quantitatively measure 27 distinct cytokines simultaneously (Table S1). Figure 2 is a heatmap with the relative quantification of all measured cytokines; the dendrogram on the left shows the clustering of cytokines with similar trends across all sample types. It can be observed that seven distinct clusters are apparent as numbered on the righthand side of the image. Cluster 1 VEGF and FGF-basic; cluster 2 IL-1ra and IL-1b; cluster 3 IL-5, IL-2, IL-9, PDGF-bb, IL-7, IFN-g and IL-17A; cluster 4 Eotaxin and G-CSF; cluster 5 MIP-1b, IL-12, IL-10, IL-4, MIP-1A, IL-13, GM-CSF and IL-15; cluster 6 RANTES, TNF-A-a and IP-10; and cluster 7 as a distal cluster made up of IL-8, MCP-1 and IL-6.

### 2.3. Cytokine Content Changes across Treatments

**Figure 3:**
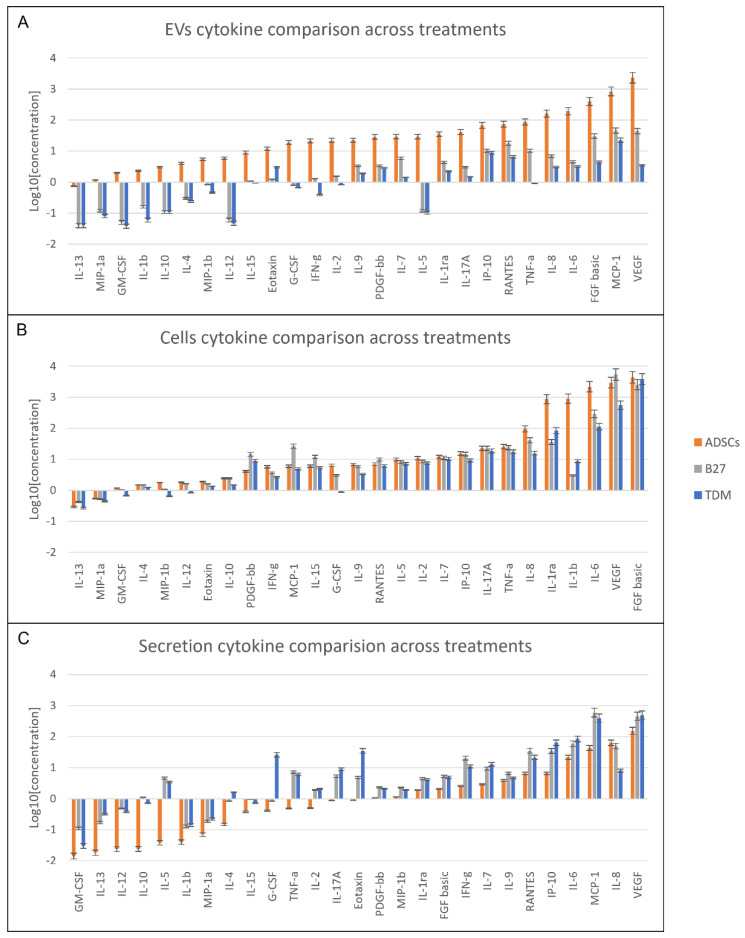
Cytokine profiles ordered in ascending concentration correlative to control ADSCs (**A**) EV (**B**) Cells (**C**) secretion based ordered cytokine profiles for control ADSCs, B27 and TDM treated cells presented in log10 of average mean values. Graph comparison order based ascending values of ADSCs.

The breakdown analysis of cytokine differences in each sample type content allows for a detailed view of the relative changes occurring between the untreated day 0 control and the day 28 treated cells. In Figure 3, the 27 cytokines are displayed in sample-specific column graphs of EVs (Figure 3A), cells (Figure 3B) and secretions (Figure 3C) are displayed as a log10 of mean values. The comparison order is based on the ADSCs control in ascending order. This graphical layout contextualises the variations in cytokines in a sample type across the various treatments. It also shows the difference in cytokine level changes and order across the sample types. The ADSC-EVs (Figure 3A) have a predominant level over their differentiating counterparts. Whilst an overall decrease is apparent across the differentiating cells’ EVs, a distinct decrease pattern is seen where IL-13, MIP-1a, GM-CSF, IL-1b, IL-10, IL-4, MIP-1b, IL-12 and IL-5 have a sizeable downshift in quantified EV cargo. In the cells, intracellular content (Figure 3B) showed a marked increase in PDGF-bb, MCP-1, IL-15, RANTES and VEGF only in the B27 treated cells. Whereas for the TDM treated cells, the contents remained lower than the control ADSCs across the board and only higher in concentration than the B27 cells for IL-1ra, IL-1b and FGF-basic. The secretion profile (Figure 3C) post B27 and TDM treatment show a vastly different profile shift from control ADSCs. The secretions display the greatest changes with cells treated with B27 and TDM, with an overall increase in cytokine expression across the entire panel of 27-cytokines. This supports the dendrogram clustering, where the B27 values are consistently higher compared to control and TDM. Except for the standout, IL-4, G-CSF, IL17A and Eotaxin showed vastly increased measures, and IL-6 and VEGF were slightly ahead for an overall increase in TDM secretion. The decrease in cytokines post-treatment appears to be a function of the treatment and differentiation process. Cytokine production and secretion profiles also change during the treatment regime.

### 2.4. Pathway Analysis of GO Clustered Cytokines with Neural Related Roles

**Figure 4:**
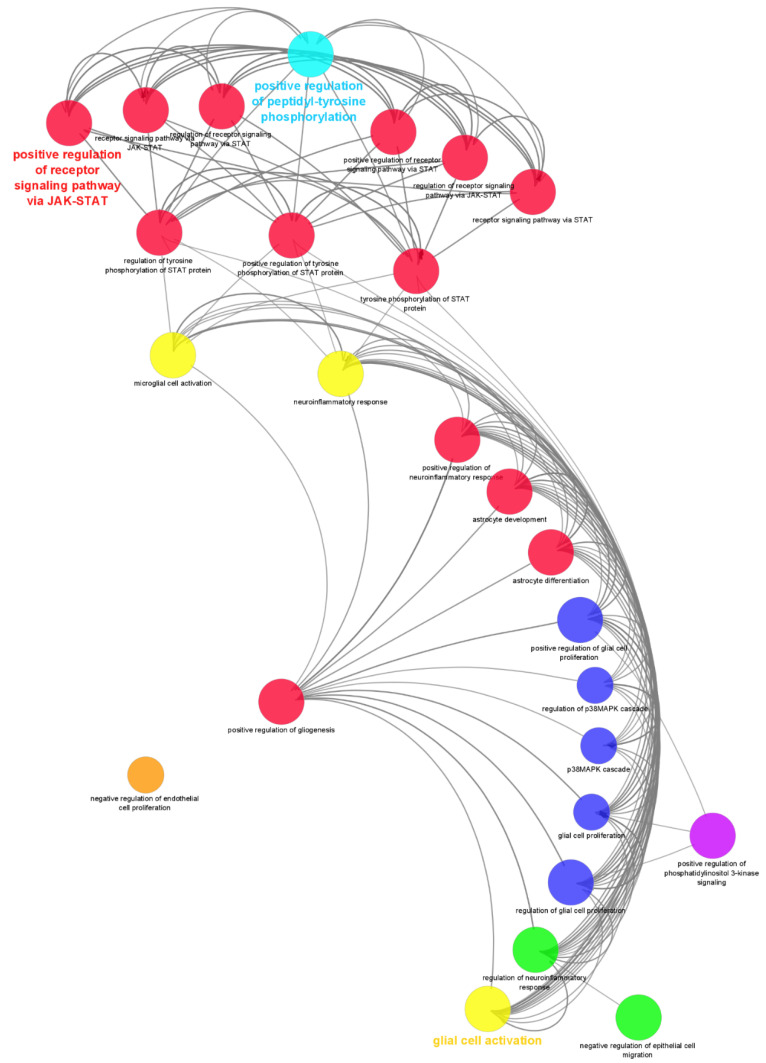
Gene ontology interaction network of the ClueGO analysis of clustered cytokines within each GO node for biological process interaction and signalling pathway analysis involved in the treated cells. Node sizes are relative to the number of proteins in the dataset identified in that GO. Coloured nodes and labels present important GO terms in-network. Red nodes indicate GO related to cell regulation and differentiation, yellow glial cell activation and neuroinflammatory response, dark blue nodes regulation of cascades, green nodes regulation of negative inflammatory response and epithelial cells, purple nodes positive regulation of phosphatidylinositol 3 kinase signalling, light blue nodes positive regulation of peptidyl-tyrosine phosphyrylation, orange nodes negative regulation of endothelial cell proliferation.

**Figure 5:**
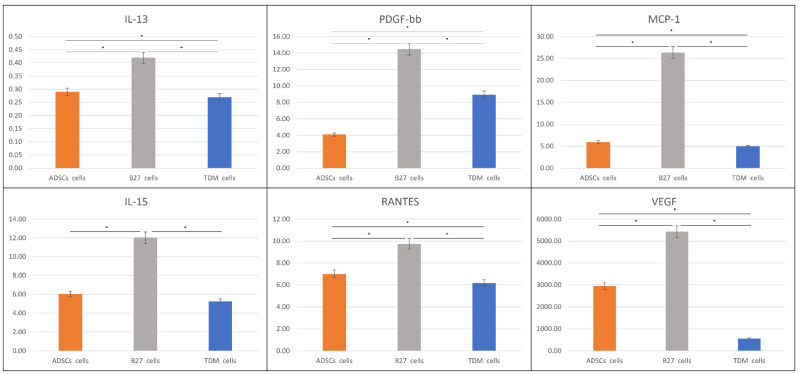
Cytokines with increased expression in B27 treated ADSCs. ADSC control (orange), B27 treated cells (grey) and TDM treated cells (blue). All concentrations in pg/mL (*y*-axis) were measured in biological triplicates by the Bioplex system. * *p* < 0.05 according to Student’s *t*-test.

The quantified cytokines were subject to gene ontology biological process analysis in ClueGO (version 2.5.8) for group clustering and interaction pathway process analysis (Figure 4). Graphing only the neural-related and signalling nodes allowed for a concise interaction map of the probable roles played in the differentiated process as well as links in signalling pathways. The network is made up of 26 nodes with 154 edge interactions with an average of 10.160 node neighbours. Table 1 break down the gene ontology types and number of proteins identified in each GO term with p-value Bonferroni step down to validate multiple pairwise tests. Figure 5 show the cytokines IL-13, PDGF-bb, MCP-1, IL-15, RANTES and VEGF, with the highest increased overall expression in only the B27 treated ADSCs intracellular space relative to all other sample types.

## 3. Discussion

Mesenchymal stem cells (MSCs) were a substantial topic of research in a variety of areas during the last decade [4,7,30]. The key areas of interest tend toward understanding the molecular mechanisms driving differentiation and maturation as well as translation to clinical applications [4,31]. The utility of accessing relatively low invasive tissue isolates containing a wealth of primary autologous stem cells holds significant value for medical research and development [32]. The variety of potential neural applications these cells and their directed differentiated counterparts possess is considerable [33]. In vitro studies have explored the effect of differentiation cocktails, growth factors [34,35] and chemicals [8,9,10,36] on MSCs, slowly expanding the prospects of and understanding from the cellular to the molecular level [37,38]. The traditional reductionist approach of investigating individual molecular outcomes from treated cells are adequately being replaced by a holistic approach in the post-omics era of research investigating a collective of subcellular components in a systems biology approach to gain a completionism view [1,39]. In this study, we aimed to observe, measure and compare the effect of two commercial neural differentiation media types on MSC cytokine modulation and expression over chosen time points across three specific sample types; intracellular, extracellular vesicles and secretions; to further understand the role this diverse group of molecules has in differentiation.

### 3.1. Morphology of Differentiating Cells

Live microscopy viewing of cellular morphology during temporal differentiation provides a direct comparison of cells over time in various treatment regimens, allowing the identification of key features and visual checks for overall cellular health and differentiation status [40]. The standout observation from the morphological changes in the B27 treated cells is the abundant and consistent progression from the typical small fibroblastic shaped MSCs with irregular growth patterns to elongated cell bodies with fine dendrite-like filaments extending from the polar regions between cells, not too dissimilar from immature neural junction formations. The other standout feature is the defined and higher refractive halo present bordering the central region of the cells, indicating a much denser and more contracted cell body. The sub-confluence at 28 days post-treatment would tend to indicate that the cells were approaching a maturation stage, with a slower proliferation rate compared to the ADSCs in control media for 28 days. The differentiating cells remained at a relatively stable number. The maturation timeline is subject to further investigation as this will require substantially longer timeframes to pinpoint the post-mitotic phase. Whilst the TDM treated cells appeared similar to the B27 treated cells, their main cell bodies were noticeably flatter and more diffused, lacking the refractive contracted central cell body. The TDM treated cells also presented a widespread neurite-like filament projection between cells. The cell numbers in both treatments are similar to each other; however, when compared to the controls at time point zero, a statistically significant increase is observed.

### 3.2. Cytokine Roles in Neural Differentiation Media Treated ADSCs

The mounting evidence of MSCs capability to differentiate toward neural-like lineage is substantial with a thematic link of an assisted or directed induction with a stimulus media. Commercial differentiation media kits and formulations boast a wide user base with a varied range of stem cell types. Gibco’s B27-supplement in neurobasal media was initially intended as a neural cell support media; the literature indicates its comprehensive use in neural differentiation in embryonic stem cells [41,42], iPSC [43,44] and MSCs [10,45,46] derived from a host of adult tissues. This increased its versatility for research, exploring the effects on cells in terms of their produced signalling molecules and roles during neural differentiation of MSCs. The TDM composite cocktail with additive growth factors increased the flexibility of media-induced differentiation, producing defined, matured cell types most notably seen in iPSCs [47]. In the intracellular content of the B27 treated cells compared to control ADSCs (Figure 5), there is a marked increase and statistical significance in IL-13, PDGF-bb, MCP-1, IL-15, RANTES and VEGF. Notably, there is no significant change in measured concentrations between IL-4 and IL-10 in B27 treated ADSCs and controls.

IL-13′s defined classical function as a regulator of Th2 cells mediates its production of IgE [48]. However, its expression in non-immune stem cells displays a multifunctional role in local anti-inflammation and signalling modulation that directs self-renewal and differentiation [49]. Interestingly the structurally related cytokine IL-4 [50], which remained unchanged between B27 and the control, shares a receptor activation functionality with IL-13. The GO graph in Figure 4 supports the notion that their signalling is directed via the IL-13 receptor activating the JAK/STAT pathway. The JAK/STAT pathway is known to be critical in the differentiation of neural stem cells [51]; it was also found to be vital in neuroprotection [52]. The neuroprotective role of IL-13 was also found to maintain neuronal integrity and synaptic function in multiple-sclerosis patients [53]. Supporting the IL-13 neuroprotective role was a study by Le Blon et al. regarding MSCs over-expressing IL-13, where it was found to reduce loss of microglia oligodendrocytes and reduce demyelination on neurons in a mouse model [54].

The expression of PDGF-bb in the B27 treatment is approximately double that measured in the controls; furthermore, the TDM treatment also shows an approximate 40% increase in expression. PDGF-bb function, as a growth factor, has varied roles, with it being most prominently known for development, cell proliferation, migration and angiogenesis [55]. Similar to IL-13, it is also known to have a neuroprotective role, particularly during oxidative stress [56]. Moreover, there is physiological evidence for the neuroprotective role of PDGF-bb in other cases of disease or injury [57]. The duality of neuroprotective molecules also has neurotrophic effects that are integral to the differentiation process [58]. PDGF-bb can induce the partial differentiation of NSCs in the absence of all other inducers, and in the presence of other additives, differentiation to neurons, astrocytes and oligodendrocytes were observed by Elandsson et al. [59]. This study also found that the expression levels of PDGF-bb were highest earlier in the neural differentiation process [59]. While MSCs undergoing neural differentiation are shown to express increasing levels of PDGF-bb, a study with MSCs pre-treated with PDGF-bb also showed increased viability and nerve regeneration. This may indicate that the secreted expression of this molecule may also affect proximal cells in vitro and in vivo, supporting the notion of localised neuroprotection during traumatic brain injury [60].

Neurotrophic and neuroprotective roles also extend to IL-15 with elevated intracellular levels which were only observed in the B27 treated cells. Interestingly, the TDM final levels were lower than the control ADSCs. From an intracellular perspective, IL-15 has a substantial role in cascade activation with fulcrum modulatory effects in JAK/STAT or MAPK pathways that are vital to proliferation and differentiation. IL-15 inflammatory function also plays an important role in neuroinflammation, having a guiding role in neuroplasticity and neurogenesis [61]. Increased intracellular levels of IL-15 have mitogenic properties seen in studies with neural stem cells where IL-15 deficiency decreased neuroblast differentiation and maturation [62]. Observing these similar trends in our studies’ cells is supported by the complementary cytokine and growth factor expression that expounds the growing evidence of a multifaceted cellular change in response to differentiation media.

The remaining molecules, MCP-1, RANTES and VEGF, have an increased expression in only the B27 treated cells. The literature indicates that mature neural cells utilise these factors in the neuroprotective route as well as to promote the maturation of specific neural cell types. MCP-1 are noted to have the associated neuroprotective effects when directly expressed in perturbed astrocytes and activated microglia [63,64]. The other interesting functionality of this molecule relates to its first established role and namesake, a chemoattractant. Neural cell expression of MCP-1 acts as a directional growth chemoattractant between cells, specifically involved in neuron and microglia interaction [65]. This is also confirmed to induce migration and directional growth in adult neural stem cells [66]. In this study, migratory and directional growth is observed at 14 days in the B27 and TDM treated cells, with advanced cellular polarisation exhibiting concurrent cell linkages in the B27 treated cells at 28 days. The consolidation of cellular morphology growth pattern is also supported by the increased expression of RANTES in the B27 treated cells. RANTES, a chemokine for T cells, is also implicated in promoting neuromodulation of synaptic activity and not surprisingly, neuroprotection [67]. The RANTES-mediated control of the principal central nervous system neurotransmitter, glutamate, is released at synapses. This may assist in neural communication as well as promoting chemotaxis of differentiating and sensory neuronal cells [68,69]. The final pleiotropic cytokine VEGF is known to have angiogenic properties; it also prevents neuron death in hypoxic and increased stress conditions imbuing both neurotrophic and neuroprotective properties [70]. The neurotrophic properties induce the PI3K/Akt transduction system that reduces programmed cell death and apoptosis in ischemic injury [71]. In summary, the retinue cluster of upregulated intracellular cytokines has a clear theme of neuroprotection and neurotrophic links. The collective analyses also reveal a clear distinction between the neural cell types promoted; this will be covered in detail in the next section.

### 3.3. Cytokines in EVs and Secretions Roles in Signalling and Differentiation

Cellular communication between cells is an intricate network of molecules that are transferred to immediate neighbours and those that are shuttled further afield across tissues, organs or systemically. This is achieved either by active, passive or assisted travel for a range of molecules that can be secreted directly to the extracellular space or packaged as cargo within extracellular vesicles (EVs) to conduct the array of molecular crosstalk between cells. In this study, both EVs and secreted material produced directly from cells into surrounding media were examined in parallel to the intracellular cytokine content.

The measured EV cytokines are an abundant collection with noticeable clusters of growth factors, chemokines and some subsets of interleukins across the control ADSCs. Post B27 and TDM treatments show a sizeable decline across the entire panel. The standout clusters with concentrations dropping to the lowest detectable range are IL-13, MIP-1a, GM-CSF, IL-1b, IL-10, IL-4, MIP-1b, IL-12, IL-15 and IL-5. Interestingly the cytokines measured from the B27 sample EVs have less downregulation compared to the TDM samples, except for Eotaxin. Eotaxin’s final concentration in the TDM samples is more than double that seen in the B27 samples. Interestingly this trend is matched in the secretion samples but not in the intracellular samples. Few studies have explored the role Eotaxin has in these cells. Current indications point to a hypothesis that an increased Eotaxin levels in the brain may decrease neurogenesis and inhibit early differentiation [72,73]. Thus, the decrease in Eotaxin in this study conforms to these findings.

While not all cytokine changes are statistically significant, minor modulations in concentrations can lead to complex biological significance downstream, particularly in signalling systems during differentiation. While it is not possible to explicate all roles for each individual cytokine change, a group cluster gene ontology analysis benefits here for clarity. The gene ontology analysis provides a clear snapshot of the complexity of the various roles and dynamics cytokines play in signalling and differentiation. Exploring their roles in differentiation, the central hub node in the network was found to be ‘glial cell activation’, where several subsidiary cytokines were all upregulated. The interacting nodes hold substantial relevance for the types of differentiation and signalling that occurs during the treatment phases. The node ‘cell differentiation’ has 22 of the 27 measured cytokines involved within it. Interacting ontology nodes indicate directing the differentiation of towardspecific neural cell types of glial cells. Interestingly there were no neuronal ontologies observed.

The increase in positive regulation of peptidyl-tyrosine phosphorylation ontology (Figure 4) is important for the regulation of the STAT3 protein that in turn plays an essential role in signalling during differentiation [74]. STAT3 and IL-6, were also substantially upregulated in the treated samples and were able to activate the surface receptor gp130 [75]. Neural stem cells’ differentiation fate is often regulated by STAT3 activation and the consequential downstream effects [76]. In stem cells and the developing brain STAT3, mediated signalling is one of the main mechanisms that promote astrocyte differentiation while suppressing neurogenesis [77,78]. This is supported by a previous study in our lab where valproic acid treatment of stem cells inhibited the JAK/STAT signalling pathway while increasing the MAPK cascade, thus promoting the development of neuronal cells [10]. Consolidating this finding within this study, the outer nodes show a downregulation of the p38MAPK cascade in response to the positive regulation of glial cell and astrocyte development nodes. The secreted upregulated cytokines clustered in astrocyte activation, development and differentiation contain several cytokines, of which two, IL-1b and IL-6, have a converse relationship with MAPK cascade. Furthermore, the multiple positive regulatory nodes promoting the signalling pathway via JAK/STAT regulation supports the duality of astrogliogenesis versus neurogenesis.

In summary, this study treated ADSCs with two commercial differentiation media formulations and examined the cytokines within the cells, secretions and in EVs at the chosen differentiation endpoints comparing these to the undifferentiated control. The observed intracellular and extracellular changes across the litany of modulated cytokines supports the findings that both ADSCs treatments promote differentiation toward astrogliogenesis. This is further confirmed by direct live cell imaging, where cells have a distinct and sizeable shift in shape and structure. The molecular analysis heatmap clustering, gene ontology analysis and the interaction network topography visually summarise these comparative findings across the various sample types. The upregulation of key cytokines intracellularly promotes neuroprotective and neurotrophic factors that are integral during the stress of morphological changes imbued during differentiation. Furthermore, the cytokines and chemokines secreted from the cells may play a sizeable role in instigating a signalling shift and promoting the JAK/STAT cascade that, in turn, favours astrogliogenesis differentiation. Whilst they are small molecules, cytokines play an enormous role in the regulation, maintenance, signalling, differentiation and cellular fate. While secreted and intracellular cytokine effects are being studied extensively, the effects of transmission through extracellular vesicles are only just becoming more understood. Their pleiotropic and multifunctional nature is slowly being unravelled. The targets and effects of cytokine expression by the various transmission pathways are the subject of future research.

Future studies may also investigate extended timepoints with targeted surface marker quantification to investigate the extent of differentiation and maturation achieved in this system. These studies may lay the foundations for refined translational applications toward modulating and promoting neural repair in situ for traumatic injury or degenerative disease patients.

## 4. Materials and Methods

### 4.1. Cell Culture

Adult human ADSCs isolation and expansion procedures were outlined by Santos et al. [79] utilising cells cryostored from UTS-HREC Santos-2013000437 ethics approval. Donor participants volunteered material through informed consent, as outlined in ethics guidelines, and were de-identified for research purposes. ADSCs were seeded at 1000 cells/mm^2^ in triplicate (n = 3) in separate T175 flasks (Nunc, ThermoScientific, Carlsbad, CA, USA) in a media mixture comprised of DMEM Glutmax/F12 (Gibco, Life Technologies, Carlsbad, CA, USA) with 10% Foetal Bovine Serum (FBS, Gibco, Life Technologies, Carlsbad, CA, USA) incubated at 37 °C at 5% CO_2_. ADSCs media was aspirated and replaced every 84 h, and cells were passaged 3–5 times by stripping cells with TrypLE Express (12604 Gibco) before being used in differentiation experiments. Cells were maintained to 80% prior to commencing differentiation experiments. Cells were counted with an automated cell counter, Countess 2 (ThermoScientific, Carlsbad, CA, USA), as per the manufacturer’s instructions.

### 4.2. Differentiation Media and Treatment

Sub-confluent ADSCs were washed twice in pre-warmed sterile D-MEM/F12 (Gibco, Life Technologies, Carlsbad, CA, USA); this was then replaced with the differentiation media B27 or terminal differentiation media (TDM). The B27 media was comprised of Neurobasal media (Gibco, Life Technologies, Carlsbad, CA, USA) supplemented with B27 (Gibco, Life Technologies, Carlsbad, CA, USA) and cultured for 28 days with media aspiration and replacement every 84 h. TDM was comprised of DMEM Glutmax/F12 (Gibco, Life Technologies, Carlsbad, CA, USA), 2% B27 (Gibco, Life Technologies, Carlsbad, CA, USA), 1% N2 (Gibco, Life Technologies, Carlsbad, CA, USA), 20 ng/mL BDNF (Peprotech, Cranbury, NJ, USA) and cultured for 28 days with media aspiration and replacement every 84 h. Then, cells were harvested post-EV and secretion collection by washing adhered cells in PBS then stripping with TrypLE Express (12604 Gibco). Cell counts were completed using Countess 2 (Thermo Fisher Scientific, NSW, Australia) according to the manufacturer’s guidelines. Harvested cells were then stored at −80 °C until sample preparation.

### 4.3. Extracellular Vesicle Isolation

EVs were isolated as similarly outlined in Dalla et al. [26], where growth media was collected from each cell treatment, and EVs were isolated by differential centrifugation. Briefly, media from each cell sample was centrifuged at 20,000× *g* for 1 h at 4 °C to pellet EVs. The pellet was then resuspended in 1× sterile phosphate-buffered saline (PBS) (Sigma-Aldrich, NSW, Australia) and centrifuged at 2000× *g* for 1 min to remove debris. The supernatant was centrifuged again at 22,000× *g* for 30 min at 4 °C to pellet EVs. The EVs were resuspended in PBS and stored at −80 °C until sample preparation. EV concentrations were determined by protein content using a Qubit protein assay (Thermo Fisher Scientific, Waltham, MA, USA) using the manufacturer’s protocol.

### 4.4. Secretion Isolation

The conditioned media from each cell sample was collected post EV isolation. Once EVs were pelleted, 500 μL of supernatant was collected from each sample and stored at −80 °C until sample preparation.

### 4.5. Sample Preparation

All samples were retrieved from −80 °C and thawed on ice. ADSC and EV samples were prepared in the same manner were pellets stored in PBS were centrifuged for 10 s at 10,000× *g,* then lysed to release inner cytokines using a probe sonicator (Sonics & Materials, Inc., Newtown, CT, USA) 3 times with 10 s bursts each while on ice. Lysed ADSCs and EVs were then centrifuged at 20,000× *g* for 10 min to remove debris, collecting supernatant in fresh Eppendorfs for analysis. Secretion samples were also centrifuged at 20,000× *g* for 10 min, collecting supernatant in fresh Eppendorfs for analysis.

### 4.6. Cytokine Assay

Bioplex analysis was performed as per Santos et al. [10] using the manufacturer’s guidelines. Generally, 50μL of prepared samples from the ADSCs, EVS and secretion’s final volume were used from each biological replicate to determine concentrations of IL-1ra, IL-1b, IL-2, IL-4, IL-5, IL-6, IL-7, IL-8, IL-9, IL-10, IL-12 (p70), IL-13, IL-15, IL-17, Eotaxin, FGF basic, G-CSF, GM-CSF, IFN-γ, MCP-1, MIP-1a, MIP-1b, PDGF-bb, RANTES, TNF-A-α and VEGF simultaneously using the commercially available multiplex bead-based sandwich immunoassay kits in triplicate (n = 3) (Bioplex human 27-plex, M50-0KCAF0Y Bio-Rad Laboratories, Hercules, CA, USA). Statistical differences were calculated by student’s *t*-test where the *p*-value was less than 0.05. The concentration was normalised relative to sample cell number.

### 4.7. Data Analysis

Data analysis was completed using Microsoft Excel 365, DanteR software (DanteR version 1.0.0.10. R version 2.12.0 The R Foundation for Statistical Computing, Auckland, New Zealand) [21] and Cytoscape (version 3.9.0 Cytoscape Consortium, Seattle, WA, USA) [80].

## Figures and Tables

**Table 1:** Gene ontology of cytokines associated in neural-related ClueGO interactions and signalling network map. Statistical significance by *p*-value < 0.05 is denoted with “*”.

GO Term	GO:ID	Number of Proteins	Associated Proteins Found	Percentage of Associated Proteins	Term *p*-Value
astrocyte activation	GO:0048143	4	[IFN-G, IL1B, IL6, TNF-A]	16.00	4.85 × 10^−6^ *
astrocyte development	GO:0014002	4	[IFN-G, IL1B, IL6, TNF-A]	8.70	4.55 × 10^−5^ *
astrocyte differentiation	GO:0048708	4	[IFN-G, IL1B, IL6, TNF-A]	4.55	3.28 × 10^−4^ *
cell development	GO:0048468	11	[EOTAXIN, MIP-1A, IFN-G, IL15, IL1B, IL2, IL5, IL6, PDGFB, TNF-A, VEGFA]	0.48	1
cell differentiation	GO:0030154	22	[EOTAXIN, MCP1, MIP-1A, GM-CSF, G-CSF, IP-10, FGF2, IFN-G, IL10, IL12A, IL13, IL15, IL17A, IL1B, IL2, IL4, IL5, IL6, IL7, PDGFB, TNF-A, VEGFA]	0.49	1
cell surface receptor signalling pathway	GO:0007166	27	[EOTAXIN, MCP1, MIP-1A, MIP-1B, RANTES, GM-CSF, G-CSF, IP-10, IL-8, FGF2, IFN-G, IL10, IL12A, IL13, IL15, IL17A, IL1B, IL1RN, IL2, IL4, IL5, IL6, IL7, IL9, PDGFB, TNF-A, VEGFA]	0.80	1
cellular developmental process	GO:0048869	22	[EOTAXIN, MCP1, MIP-1A, GM-CSF, G-CSF, IP-10, FGF2, IFN-G, IL10, IL12A, IL13, IL15, IL17A, IL1B, IL2, IL4, IL5, IL6, IL7, PDGFB, TNF-A, VEGFA]	0.48	1
central nervous system development	GO:0007417	4	[IFN-G, IL1B, IL6, TNF-A]	0.36	1
chemokine-mediated signalling pathway	GO:0070098	7	[EOTAXIN, MCP1, MIP-1A, MIP-1B, RANTES, IP-10, IL-8]	7.78	7.34 × 10^−9^ *
glial cell activation	GO:0061900	7	[MIP-1A, IFN-G, IL13, IL1B, IL4, IL6, TNF-A]	11.86	3.77 × 10^−10^ *
glial cell development	GO:0021782	4	[IFN-G, IL1B, IL6, TNF-A]	3.25	1
glial cell differentiation	GO:0010001	4	[IFN-G, IL1B, IL6, TNF-A]	1.68	1
glial cell proliferation	GO:0014009	3	[IL1B, IL6, TNF-A]	5.66	1.01 × 10^−3^ *
MAPK cascade	GO:0000165	14	[EOTAXIN, MCP1, MIP-1A, MIP-1B, RANTES, GM-CSF, FGF2, IL1B, IL2, IL5, IL6, PDGFB, TNF-A, VEGFA]	1.44	1
negative regulation of cell population proliferation	GO:0008285	10	[MCP1, IL-8, IFN-G, IL10, IL12A, IL15, IL1B, IL2, IL6, TNF-A]	1.33	1
negative regulation of endothelial cell proliferation	GO:0001937	3	[MCP1, IL12A, TNF-A]	6.98	7.89 × 10^−4^ *
negative regulation of epithelial cell differentiation	GO:0030857	3	[IFN-G, IL13, VEGFA]	6.00	9.61 × 10^−4^ *
neuroinflammatory response	GO:0150076	7	[MIP-1A, IFN-G, IL13, IL1B, IL4, IL6, TNF-A]	9.59	1.72 × 10^−9^ *
neurotransmitter metabolic process	GO:0042133	4	[IFN-G, IL10, IL1B, TNF-A]	2.48	1
p38MAPK cascade	GO:0038066	3	[IL1B, IL6, VEGFA]	6.25	9.00 × 10^−4^ *
positive regulation of cell differentiation	GO:0045597	17	[MIP-1A, GM-CSF, G-CSF, FGF2, IFN-G, IL12A, IL13, IL15, IL17A, IL1B, IL2, IL4, IL5, IL6, IL7, TNF-A, VEGFA]	1.60	1
positive regulation of glial cell proliferation	GO:0060252	3	[IL1B, IL6, TNF-A]	17.65	1.16 × 10^−4^ *
positive regulation of gliogenesis	GO:0014015	4	[MIP-1A, IL1B, IL6, TNF-A]	5.06	2.42 × 10^−4^ *
positive regulation of nervous system development	GO:0051962	7	[MIP-1A, IFN-G, IL1B, IL2, IL6, TNF-A, VEGFA]	1.17	1
positive regulation of neurogenesis	GO:0050769	7	[MIP-1A, IFN-G, IL1B, IL2, IL6, TNF-A, VEGFA]	1.33	1
positive regulation of neuroinflammatory response	GO:0150078	4	[MIP-1A, IL1B, IL6, TNF-A]	26.67	6.21 × 10^−7^ *
positive regulation of peptidyl-serine phosphorylation	GO:0033138	5	[G-CSF, IFN-G, IL6, TNF-A, VEGFA]	4.03	6.70 × 10^−5^ *
positive regulation of peptidyl-tyrosine phosphorylation	GO:0050731	14	[RANTES, GM-CSF, G-CSF, IFN-G, IL12A, IL13, IL15, IL2, IL4, IL5, IL6, PDGFB, TNF-A, VEGFA]	6.51	2.83 × 10^−18^ *
positive regulation of phosphatidylinositol 3-kinase signalling	GO:0014068	5	[RANTES, G-CSF, IL6, PDGFB, TNF-A]	5.00	2.90 × 10^−5^ *
positive regulation of receptor signalling pathway via JAK-STAT	GO:0046427	12	[RANTES, GM-CSF, IFN-G, IL10, IL12A, IL13, IL15, IL2, IL4, IL5, IL6, TNF-A]	12.90	5.90 × 10^−19^ *
positive regulation of receptor signalling pathway via STAT	GO:1904894	12	[RANTES, GM-CSF, IFN-G, IL10, IL12A, IL13, IL15, IL2, IL4, IL5, IL6, TNF-A]	12.50	8.79 × 10^−19^ *
positive regulation of tyrosine phosphorylation of STAT protein	GO:0042531	10	[RANTES, GM-CSF, IFN-G, IL12A, IL13, IL15, IL2, IL4, IL6, TNF-A]	13.89	8.61 × 10^−16^ *
receptor signalling pathway via JAK-STAT	GO:0007259	13	[MCP1, RANTES, GM-CSF, IFN-G, IL10, IL12A, IL13, IL15, IL2, IL4, IL5, IL6, TNF-A]	7.69	1.01 × 10^−17^ *
receptor signalling pathway via STAT	GO:0097696	13	[MCP1, RANTES, GM-CSF, IFN-G, IL10, IL12A, IL13, IL15, IL2, IL4, IL5, IL6, TNF-A]	7.47	1.48 × 10^−17^ *
regulation of cell development	GO:0060284	9	[EOTAXIN, MIP-1A, IFN-G, IL1B, IL2, IL5, IL6, TNF-A, VEGFA]	0.87	1
regulation of cell differentiation	GO:0045595	20	[EOTAXIN, MIP-1A, GM-CSF, G-CSF, IP-10, FGF2, IFN-G, IL12A, IL13, IL15, IL17A, IL1B, IL2, IL4, IL5, IL6, IL7, PDGFB, TNF-A, VEGFA]	1.01	1
regulation of glial cell proliferation	GO:0060251	3	[IL1B, IL6, TNF-A]	8.82	4.91 × 10^−4^ *
regulation of neuroinflammatory response	GO:0150077	5	[MIP-1A, IL1B, IL4, IL6, TNF-A]	14.29	2.00 × 10^−7^ *

## Data Availability

Data is contained within the article or Supplementary Material. The data presented in this study are available in Table S1.

## Supplementary Materials

The following are available online at https://www.mdpi.com/article/10.3390/life12030392/s1, Table S1. Bioplex average concentrations for ADSCs, B27 and TDM for cells, secretions and Evs.
