# Supplementary material for: Molecular Dynamics of Cytokine Interactions and Signalling of Mesenchymal Stem Cells Undergoing Directed Neural-like Differentiation
Source: Life (Basel). 2022 Mar 8;12(3):392. doi: 10.3390/life12030392 (PMC8948714; doi:10.3390/life12030392)
Supplement: Supplementary file 1 [file life-12-00392-s001.zip › life-1573570-supplementary.pdf]

Table S1: Bioplex average concentrations for ADSCs, B27 and TDM for cells, secretions and Evs

Samples	ADSCs EV	B27 EV	TDM EV	ADSCs cells	B27 cells	TDM cells	ADSCs secretions
**Eotaxin**	11.98	1.26	3.04	1.93	1.63	1.35	0.90
**FGF basic**	400.30	30.92	4.40	4395.01	2556.20	3849.90	2.06
**G-CSF**	19.16	0.79	0.67	6.37	3.01	0.87	0.41
**GM-CSF**	2.00	0.05	0.04	1.18	1.05	0.68	0.01
**IFN-g**	21.46	1.28	0.38	5.71	3.65	2.64	2.59
**IL-10**	3.00	0.11	0.11	2.42	2.42	1.50	0.02
**IL-12**	5.90	0.06	0.05	1.80	1.61	0.83	0.02
**IL-13**	0.74	0.04	0.04	0.29	0.42	0.27	0.02
**IL-15**	8.98	1.09	0.95	6.04	12.03	5.26	0.38
**IL-17A**	41.28	3.00	1.50	22.76	22.43	18.54	0.87
**IL-1b**	2.34	0.16	0.06	899.10	3.06	8.66	0.04
**IL-1ra**	35.16	4.32	2.22	869.21	36.68	83.56	1.90
**IL-2**	21.98	1.54	0.84	10.88	8.53	7.45	0.50
**IL-4**	4.04	0.30	0.24	1.50	1.50	1.22	0.15
**IL-5**	29.58	0.12	0.10	9.90	8.12	7.18	0.04
**IL-6**	192.28	4.56	3.24	2171.34	289.53	113.22	21.70
**IL-7**	29.14	5.82	1.44	12.04	11.18	10.32	2.91
**IL-8**	162.32	6.86	3.12	96.25	41.00	15.67	64.02
**IL-9**	22.34	3.38	1.94	6.73	5.86	3.39	3.87
**IP-10**	67.78	10.32	8.88	15.32	14.73	9.20	6.56
**MCP-1**	840.82	46.36	22.56	5.97	26.38	4.99	43.55
**MIP-1a**	1.18	0.12	0.08	0.54	0.52	0.44	0.07
**MIP-1b**	5.50	0.82	0.46	1.77	1.07	0.65	1.15
**PDGF-bb**	28.92	3.35	2.84	4.09	14.46	8.93	1.09
**RANTES**	73.88	17.86	6.56	7.01	9.75	6.17	6.54
**TNF-a**	87.70	10.28	0.89	25.65	23.67	17.46	0.49
**VEGF**	2307.18	43.82	3.45	2955.20	5427.55	558.64	155.46

B27 secretions	TDM secretions
4.85	35.12
5.24	4.89
0.84	26.08
0.11	0.03
20.38	11.04
1.13	0.72
0.49	0.38
0.17	0.32
0.97	0.72
5.35	8.88
0.13	0.14
4.42	4.17
1.91	2.11
0.83	1.61
4.60	3.44
59.57	84.31
9.44	12.89
49.60	8.03
6.55	4.68
34.67	64.05
601.35	400.61
0.19	0.22
2.30	1.94
2.34	2.09
35.22	21.47
7.18	6.16
455.63	499.42
